# A Robust 96.6-dB-SNDR 50-kHz-Bandwidth Switched-Capacitor Delta-Sigma Modulator for IR Imagers in Space Instrumentation †

**DOI:** 10.3390/s17061273

**Published:** 2017-06-02

**Authors:** Michele Dei, Stepan Sutula, Jose Cisneros, Ernesto Pun, Richard Jan Engel Jansen, Lluís Terés, Francisco Serra-Graells

**Affiliations:** 1 Instituto de Microelectrónica de Barcelona IMB-CNM(CSIC), 08193 Barcelona, Spain; ssutula@hotmail.com (S.S.); jose.cisneros@imb-cnm.csic.es (J.C.); lluis.teres@imb-cnm.csic.es (L.T.); paco.serra@imb-cnm.csic.es (F.S.-G.); 2 Arquimea Ingenierìa S.L.U., 28918 Madrid, Spain; epun@arquimea.com; 3 European Space Research and Technology Centre, 2201 AG Noordwijk, The Netherlands; Richard.Jansen@esa.int; 4 Department of Microelectronics and Electronic Systems of Universitat Autònoma de Barcelona, 08193 Barcelona, Spain

**Keywords:** infrared imager (IR), analog-to-digital converter (ADC), delta-sigma modulator (DSM), switched capacitors (SC), class-AB operational amplifiers

## Abstract

Infrared imaging technology, used both to study deep-space bodies’ radiation and environmental changes on Earth, experienced constant improvements in the last few years, pushing data converter designers to face new challenges in terms of speed, power consumption and robustness against extremely harsh operating conditions. This paper presents a 96.6-dB-SNDR (Signal-to-Noise-plus-Distortion Ratio) 50-kHz-bandwidth fourth-order single-bit switched-capacitor delta-sigma modulator for ADC operating at 1.8 V and consuming 7.9 mW fit for space instrumentation. The circuit features novel Class-AB single-stage switched variable-mirror amplifiers (SVMAs) enabling low-power operation, as well as low sensitivity to both process and temperature deviations for the whole modulator. The physical implementation resulted in a 1.8-mm^2^ chip integrated in a standard 0.18-μm 1-poly-6-metal (1P6M) CMOS technology, and it reaches a 164.6-dB Schreier figure of merit from experimental SNDR measurements without making use of any clock bootstrapping, analog calibration, nor digital compensation technique. When coupled to a 2048×2048 IR imager, the current design allows more than 50 frames per minute with a resolution of 16 effective number of bits (ENOB) while consuming less than 300 mW.

## 1. Introduction

High levels of miniaturization are required in space for the realization of sensitive optical and radiation detector arrays. These detector arrays are used to map the particles, as well as the photons originating from stars and planets over the whole energy spectrum with the purpose to monitor the state of vegetation and pollution on Earth, as well as investigating the physical state and functioning of our surrounding universe. From the different detector arrays for the observation of particles, X-rays, UV, visible, infrared and far infrared radiation, the imaging of near and far infrared proves to be the most challenging. These detector arrays consist of multi-millions of pixels that have to readout at increasingly higher speeds and higher accuracy at minimal power expenditure, such as to minimize the induced thermal noise. At the turn of the century, the state-of-the-art detector array technology and readout electronics [1,2] consisted of 2048×2048 detector arrays manufactured either with CCD and CMOS technology with HgCdTe detector material for the infrared radiation (IR). Common configurations of pixel readout circuitry comprise source follower (SF), capacitive transimpedance amplifier (CTIA) and direct injection (DI) depending on the flux quantity over the exposure period of time and the specific wavelength range of the application. The detector array control and signal conversion component in [3] could readout up to 36 channels in parallel. Per channel signal conversion could be realized at 100 kS/s to 14 bit accuracy per ADC for 1.3 mW power. The control and signal conversion component set the detector supply, bias voltages and supplied the detector readout clocking scheme, as well as configuration via a serial bus. This detector array with the attached readout component together with the control and signal conversion component proved to be successful and has been used in many payload missions as employed for astronomical observation on Earth.

Over the ensuing years, the scientific requirements for increasing precision and resolution led to larger detector arrays [4], higher accuracy through the suppression of the detector dark currents [5,6] and increasing dynamic range [7]. With improved readout electronic components [8], linearity could be increased and noise minimized to cover and exceed the current generation of control and signal conversion components [9,10] under development. Detector array sizes increased from 2048×2048 to 4096×4096. At the same time, the dynamic range as expressed as the ratio of the maximum electron charge to noise has increased from 14 bits to 16 bits. The increased detector size has led to an increase in readout channels that have to be processed in parallel with a minimum increase in power consumption such as to minimize the thermal noise. The increased detector signal dynamic range beyond 90 dB has to be addressed with an improved ADC, which is described in the following sections.

**Figure 1:**
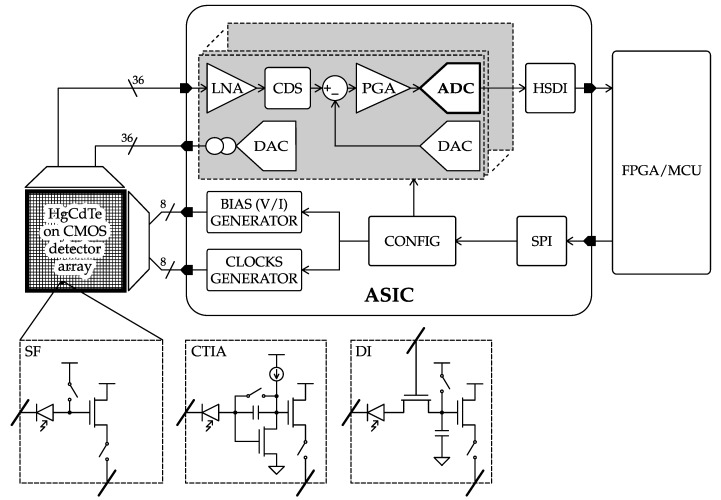
Application-specific integrated circuit (ASIC) concept for IR imagers in space instrumentation.

Typically, the component should consume less than 600 mW of power such as to allow efficient heat extraction in space. In addition to being radiation tolerant and having highly reliability, it should operate under an extended temperature range, including cryogenic temperatures down to 35K. At least 36 ADC and with each of them an offset correction DAC should be provided to shift the detector output voltage range. After offset correction, programmable gain should ensure that the full detector output signal range can be converted with the ADC. A sampler prior to the ADC should enable correlated double sampling, as well as speeding-up the readout by combining the channels via a multiplexer. The channels require also a programmable current source DAC to bias their open drain readout transistor. Moreover, each of the channels should have its own dedicated reference voltage with overcurrent and short circuit protection. The detector supply should also be provided. The house keeping ADCs should be supported with the current source to allow temperature readout from temperature sensors, as well as monitoring currents and reference voltages. In Figure 1a, a functional diagram of the detector and readout control and signal conversion component is depicted. As the detector with the readout circuit can be tiled to cover a larger surface area, the control and conversion component should not be larger in size than the detector itself.

The control and signal conversion component should provide the detector and its readout circuit with supply, bias voltages, configuration data, detector pixel reset and readout clocking signals, as well as provide the signal conditioning and conversion. In addition to these detector and readout supports, control and processing functions, it should monitor supply current, temperature and reference voltage and should transmit the collected data to the connected data processing unit through a high speed digital interface (HSDI). These operations are controlled by an on-chip sequencer that can be configured by the connected data processing unit.

The functional and operation requirements on this image detector control and signal conversion component put demanding constraints on the ADC in terms of accuracy, bandwidth, power consumption and area.

This paper presents a low-power SC ΔΣM for ADC with a 96.6-dB peak Signal-to-Noise-plus- Distortion Ratio (SNDR) and a 50-kHz bandwidth, operating at the nominal 1.8-V supply voltage of the CMOS process, and it does not require any analog calibration, DEM or digital compensation technique. This performance is mainly achieved by the introduction of Class-AB single-stage variable-mirror amplifiers (VMAs), first proposed by these authors in [11], combined with a power-aware modulator discrete-time (DT) architecture and a five-phase SC scheme. This work was introduced in [12], but extensive new material is included here concerning ΔΣM architecture selection and performance, circuit-block specifications, switched-VMA operation, ΔΣM robustness against technology mismatch, process and temperature variations and expanded state-of-the-art comparison.

The presented paper is organized as follows: Section 2 reviews the state-of-the-art ΔΣ-ADCs with emphasis on the current application. Section 3 describes the selection of the ΔΣM architecture based on power and robustness considerations. Then, the ΔΣM SC topology with switched-VMAs and a five-phase switching scheme is analyzed in Section 4, together with the amplifiers’ required performance. Section 5 proposes the Class-AB single-stage switched VMA to be used in all amplifier blocks of the noise shaper, providing a detailed analysis of its circuit operation. Section 6 presents the ΔΣM robustness against process and temperature from transistor-level simulations. Finally, the experimental results in 0.18µm-CMOS technology and a comparative analysis with state-of-the-art high-resolution ADCs are reported in Section 7.

## 2. ΔΣ ADC for Space Instrumentation

The integration of high-resolution data converters in CMOS technologies with increasing process variability and supply-voltage downscaling has become a circuit design challenge. This fact can be noticed by the small number of circuit implementations reported in the literature, like [13,14,15,16,17,18,19,20,21,22,23], compared to other regions of the ADC design universe with similar figure of merit values [24].

From the architectural viewpoint, both discrete-time (DT) and continuous-time (CT) delta-sigma modulators (ΔΣMs) are the common choice for high-resolution data converters, as they tend to relax the specifications of analog blocks at the expense of increasing oversampling ratios. Continuous-time strategies based on active-Resistor-Capacitor (RC) [13,15,18,21,23] or transconductor-capacitor (Gm-C) [19] implementations are attractive due to their built-in anti-aliasing filtering and the low-speed requirements for their active circuits. However, CT techniques can suffer from both clock-jitter and technology sensitivity, and special attention must be paid to excess loop delays in the feedback DAC. Moreover, CT noise shapers may require in practice accurate tuning circuits. On the other hand, discrete-time (DT) ΔΣM realizations relying on switched-capacitor (SC) [14,16,17,20,22] or switched-RC (SRC) [25] circuits can exploit their advantages in terms of low sensitivity to clock jitter, the high linearity of integrated capacitors and matching-based design methodologies. All of these points usually compensate for the increase of circuit area, which, in turn, can be mitigated if CMOS technologies with stacked metal-insulator-metal (MIM) or fringing capacitors are available.

Recent advances in CT shapers proved excellent power efficiency, despite the aforementioned shortcomings. On the other hand, robustness and DT shapers are essential for the required application. Hence, a deeper analysis of DT-SC shapers’ power consumption is needed. In this latter case, power consumption is identified by two contributions: static current is constantly drawn from the supplies to bias the active blocks, plus an impulsive current is needed to charge the capacitors at the pace of the clock frequency. In SC ΔΣMs, this problem is aggravated due to the oversampling nature of the modulation. Moreover, even in the condition of constant zero input, the modulator internal state variables are incessantly updated at each clock cycle due to the non-linear feedback of the system. This implies that the dynamic power consumption is practically independent of the input signal level. When operated in Class-A, the amplifiers’ bias currents must be at least on the same level as the peak impulsive currents demanded for the given settling requirements, making the static current contribution dominant over the dynamic one. To avoid this issue, active blocks operating in Class-AB are a common choice [26]. Finally a further 50% reduction of static power consumption can be achieved employing the switched-OpAmp technique [27], which consists of turning off the amplifiers during their inactive clock phase.

## 3. ΔΣ Architecture Selection

**Figure 2:**
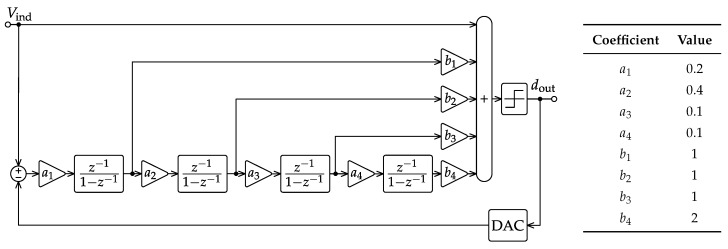
Differential-signal discrete-time (DT) model of the 1-bit 4th-order feedforward single-loop ΔΣM architecture. The $a_{1-4},b_{1-4}$ values are alike the ones used in [28].

Figure 2 presents the one-bit fourth-order feedforward single-loop ΔΣM architecture chosen for the high-resolution ADC, targeting a 16-bit dynamic range, a 50-kHz bandwidth and a 2.4-V_pp_ differential input full-scale. In this sense, the 16-bit specification is initially extended to ${\mathrm{SNDR}}_{\max}=110\;\mathrm{dB}$ in order to gain a two-bit safety margin for the rest of the ΔΣM circuit design methodology. Although Figure 2 is a behavioral DT model only, several design decisions have already been made at this level affecting its CMOS circuit implementation, especially from the power-consumption viewpoint.

The first and most noticeable design choice in the ΔΣM of Figure 2 is about the use of a single-loop architecture instead of multi-stage noise-shaping (MASH) alternatives [29]. This decision is related to the difficulty of obtaining the high-resolution analog matching between MASH stages required to benefit from the feedforward error-cancellation mechanism. In counterpart, and because of the high loop order demanded by the target dynamic range, special care must be taken to select a safe set of gain coefficients to ensure modulator stability, as explained later in this section.

Secondly, multiple feedforward paths [30] are incorporated into the ΔΣM of Figure 2. This architectural strategy allows reducing the signal components following the first integrator and investing in the high-gain integrating stages to process quantization noise mainly. As a result, specifications for the integrators’ amplifiers can be relaxed due to the lower signal content in the internal full scale. In practice, it can be shown that a reduction of circuit power consumption can be obtained when using feedforward paths for a given order of noise shaping [31].

Architectures based on multi-bit quantizers are attractive mainly because they allow one to reduce the slew rate requirements of OpAmps by lowering the amplitude of error signals inside the noise shaper with a clear benefit in terms of power consumption. Multi-bit quantization can be achieved using arrays of comparators, but circuit complexity increases exponentially with the number of bits. Power-efficient implementation of multi-bit quantization is currently a hot topic: solutions based on the successive approximation register (SAR) quantizer [15,32] or tracking quantizers [33] are popular since their power consumption scales linearly with the number of bits [32]. Alternatively, quantizers operating time-to-digital conversion (TDC) received special attention as they also promise to be very power efficient [34]. Nevertheless, to the authors knowledge, robust implementations against temperature process and voltage corners are hard to find. Finally, mismatch in the unit elements of the feedback DAC affects negatively the SNDR, and correction techniques, either calibration [13] or dynamic element matching [35], need to be employed at the cost of increased circuit complexity. Clearly, in all of the reviewed multi-bit solutions, quantizer/DAC power is traded for amplifiers’ power. In this design, we opted for the single-bit quantizer solution due to its robustness against technology mismatch, its inherent linearity and design simplicity, while power optimization is enabled thanks to the new family of Class-AB amplifiers. It must be said that the use of these special amplifiers does not prevent multi-bit solutions to be employed.

**Figure 3:**
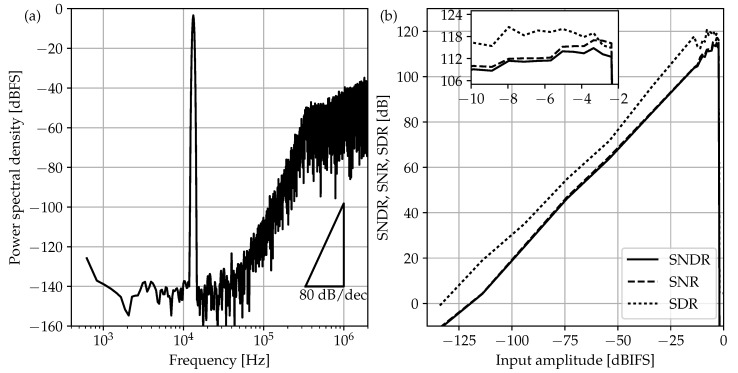
Behavioral simulation results for the ΔΣM of Figure 2 considering quantization errors and input-sampler thermal noise: output spectra for −3.35-dB_IFS_ (IFS: internal full scale) 13.28-kHz eight-cycle input with SNDR = 110 dB (**a**) and dynamic range at the same frequency (**b**).

At this point, there is a clear trade off between shaping order and oversampling ratio (OSR). Selecting a high-order shaping solution effectively lowers the OSR, so circuit speed requirements, but at the cost of increasing ΔΣM-loop instability issues. On the other hand, high OSR values require more power-efficient circuits, but they can reduce both the number of active blocks and also the area of the input sampler. Based on the 110-dB ${\mathrm{SNDR}}_{\max}$ target, a fourth-order shaping transfer function is chosen in Figure 2 combined with a moderate OSR of 136, which results in a sampling rate of 13.6 MS/s for a 50-kHz bandwidth. The behavioral simulation of the final ΔΣM architecture gives the results of Figure 3 when including the thermal noise of the input sampler. In particular, the maximum tolerated noise floor to satisfy the ${\mathrm{SNDR}}_{\max}$ specification at room temperature imposes a minimum single-ended input sampling capacitance ($C_{s1}$) exceeding 41pF.

**Figure 4:**
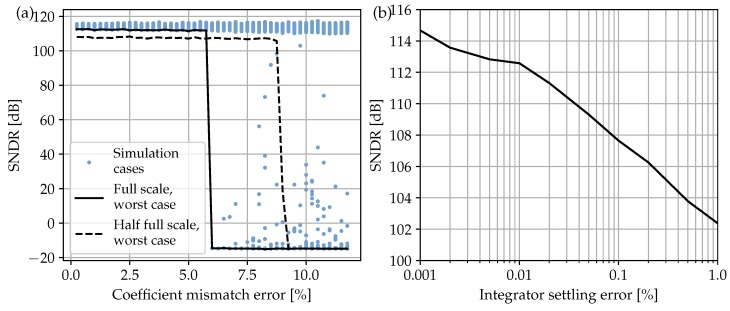
Simulated SNDR losses in the ΔΣM architecture of Figure 2 due to coefficient technology mismatch; worst case interpolation lines are given for input full scale (solid) and half full scale (dashed) (**a**); SNDR losses due to settling error at integrator outputs (**b**).

Although the chosen ΔΣM architecture differs from the latter by the absence of the $z^{-1}$ delay in the first and third stages due to the switched-OpAmp operation explained in the next section, the same set of coefficients proves the good robustness against technology mismatching up to more than 5%, as noticed from the behavioral simulation results of Figure 4a.

Transient settling errors are studied for the DT integrators of Figure 2 to foresee possible power consumption bottlenecks before starting with the actual SC circuit design of the next section. A non-linear model is built for this purpose inspired by the combined response of the amplifier slew rate (SR) and gain-bandwidth product (GBW) [36]. The results obtained from the corresponding behavioral simulation of Figure 4b reveal that a maximum settling error below 0.04% should be ensured when designing each amplifier circuit.

The digital output stream $d_{\mathrm{out}}$ in Figure 2 is finally processed by an attached FPGA, which is in charge of filtering the out-of-band modulation noise and decimating the sample rate down to the original Nyquist bandwidth of the signal. A dedicated decimator filter has not been implemented here to allow for reconfigurability with respect to different IR sensor setups.

## 4. ΔΣ Modulator SC Topology

**Figure 5:**
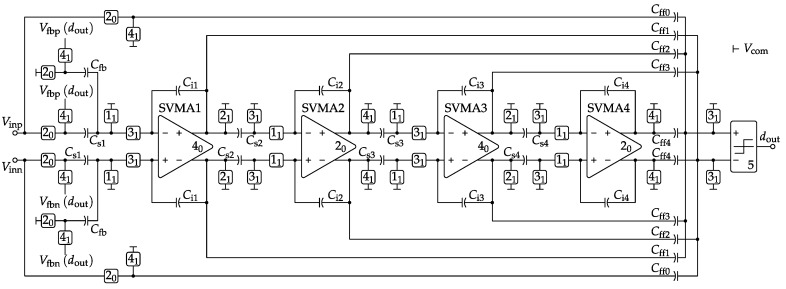
SC topology proposed for the ΔΣM architecture of Figure 2 with the switched-variable-mirror amplifier (SVMA) and five-phase switching scheme. The phase subindex indicates the special state (1 = closed, 0 = open) during ADC initialization ($d_{\mathrm{en}}=0$).

Figure 5 shows the SC network proposed for implementing the one-bit fourth-order feedforward single-loop ΔΣM architecture of Figure 2. The sizing of the sampling capacitor for each stage detailed in Table 1 is derived from the $C_{s1}$ thermal-noise specification of the previous section and the following noise-shaping scaling rule [31]:

(1) $$C_{si}=C_{s1}\frac{\pi^{2i-2}}{{\mathrm{OSR}}^{2i-2}\left(2i-1\right)}\prod_{k=2}^{i}\frac{1}{a_{k-1}^{2}}\;\mathrm{for}\;i>1,$$

where $a_{k-1}$ stands for the gain of the (k-1)-th integrator. The other capacitor sizes of Table 1 directly come from the rest of the coefficients of Figure 2. It is worth noting at this point the large capacitance values obtained for the first stage (i.e., $C_{s1}$, $C_{\mathrm{fb}}$ and $C_{i1}$). This challenging specification, which allows one to keep the signal full scale within the nominal CMOS supply voltage (1.8 V) and to avoid any bootstrapping at the input stage, demands very efficient Class-AB amplifiers for low-power operation, as proposed in the next section.

Switched-OpAmp operation is employed in all integrators of the ΔΣM. In our context, its main purpose is not only to replace the critical series switches at the output of each stage, which can introduce distortion issues due to their signal-dependent resistance, but also to save DC current consumption thanks to the 50% duty cycle of each amplifier. In this sense, the clock phase controlling the on-off state of each amplifier in Figure 5 is indicated inside its symbol following the same nomenclature as for the rest of the switching elements. It is important to highlight that all switches are implemented using simple complementary N/PMOS transmission gates and operated at the nominal supply voltage of the target CMOS technology without any clock-bootstrapping technique, which is incompatible with the high-reliability standards of space applications since it heavily overstress the gate oxide of the sampling devices, degrading the signal quality and finally shortening their lifetime [37].

As already discussed in the previous section, a passive capacitive divider is employed in Figure 5 for the summation of all feedforward signals coming from the nested paths. This solution has a double benefit in terms of low-power operation, since it avoids the DC current consumption of OpAmp-based active summers, and it relaxes the timing requirements for the quantization block. This fact, together with the low sensitivity of single-bit quantization to circuit non-idealities, such as voltage offset, enables the use of a very simple regenerative comparator circuit as the quantizer, like [38], but without the preamplifier stage. Finally, the SC implementation of the single-bit feedback DAC of Figure 5 shares some part of its capacitors with the input sampler in order to reduce the overall circuit area.

**Figure 6:**
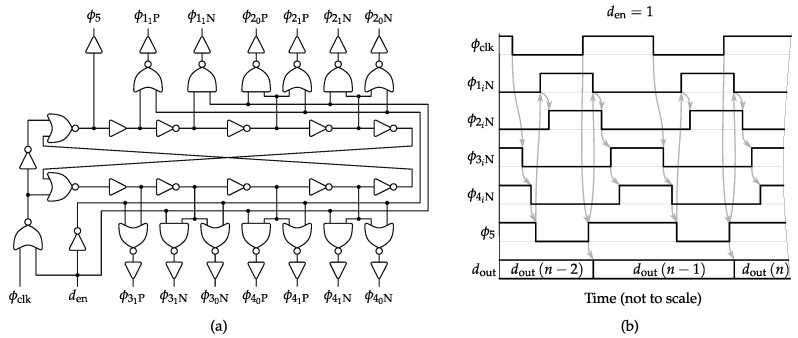
Clock phase splitter and initializer for the SC ΔΣM of Figure 5 (**a**) and five-phase switching chronogram for the NMOS driving case (**b**).

The ΔΣM phase splitter is presented in Figure 6a. It employs a specific five-phase circuit in charge of generating dedicated control signals for each NMOS and PMOS device used in the four switch locations of the SC integrators plus the quantizer. The generated sequence of Figure 6b ensures that the right-hand switches of the sampling capacitors $C_{s1-4}$ of Figure 5 are opened before their left-hand counterparts [28]. In other words, the first switch to be opened for each sampling capacitor is always connected to the common reference (i.e., differential-signal ground). In this way, when the switch connected to the other terminal of the same sampling capacitor is also opened, no signal-dependent charge can be injected in this later terminal because the former terminal is already in high impedance. As a result, no distortion components are ideally introduced in the differential sampling, but only a constant amount, which is rejected by the amplifier common mode feedback (CMFB). Concerning the input sampler and the feedback DAC switch cases, it is found that by combining minimum channel length and width optimization of purely standard complementary N/PMOS transmission gates without gate bootstrapping, the resulting sampled signal can achieve in practice SDR values above 125 dB for the target CMOS technology.

Table 2 summarizes the loading conditions and speed requirements for each particular switched VMA of Figure 5. In this sense, $C_{\mathrm{load}}$ stands for the equivalent single-ended load capacitance, while the particular feedback gain factor of the *x*-th amplifier can be expressed as:

(2) $$\beta_{\mathrm{fb}x}=\left\{\begin{array}{cc} \frac{C_{ix}}{C_{sx}+C_{\mathrm{fb}}+C_{ix}}, & x=1;\\ \frac{C_{ix}}{C_{sx}+C_{ix}}, & x>1. \end{array}\right.$$

Concerning settling time ($t_{\mathrm{sett}}$), the final specification value of Table 2 is obtained by combining the maximum allowed error from the behavioral analysis of Figure 4b together with the selected OSR and a conservative 25% of clock period reserved for the non-overlapping guard intervals of Figure 6b.

## 5. Class-AB Switched-VMA Circuit

**Figure 7:**
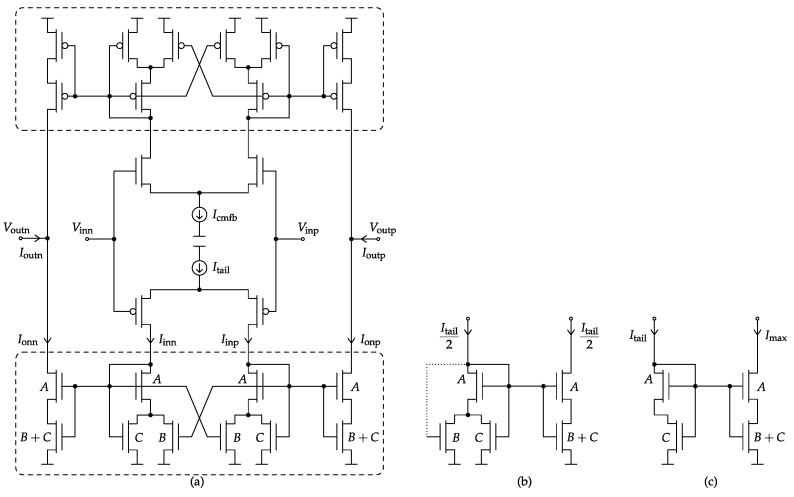
Simplified topology of the Type-II Class-AB single-stage VMA (**a**); equivalent right-half circuit of the NMOS boxed path in the DC operating point $I_{\mathrm{inp}}\equiv I_{\mathrm{inn}}\equiv I_{\mathrm{tail}}/2$ (**b**) and for the maximum Class-AB positive output when $I_{\mathrm{inp}}≃$ and $I_{\mathrm{tail}}$$I_{\mathrm{inn}}≃0$ (**c**); the dotted line in (**b**) indicates a virtual short circuit.

Figure 7a presents the low-power Class-AB single-stage VMA topology employed for each amplifier of the SC ΔΣM of Figure 5. In what follows, all MOSFET bulk terminals are connected to their respective supply rail. The VMA Class-AB control, first introduced by these authors in [11] as Type-II, is split into the two boxed paths of Figure 7a for the individual and symmetrical control of the NMOS and PMOS output transistors. The full topology can be understood as a variable-gain current mirror (*A*-size devices) with a non-linear voltage control (*B*- and *C*-size devices). Alternatively, it can be classified as a source-degeneration technique [39], but with the novelty of being dynamic and specifically designed to cancel process and temperature dependencies.

The core of the VMA topology of Figure 7a consists of the *B*-sized cross-coupled matched pair introduced here to supply the local positive feedback responsible for emphasizing the Class-AB behavior. However, in order to prevent from an excess of positive feedback gain, which would otherwise latch the entire amplifier circuit, an additional *C*-sized pair is attached. In practice, the optimum balance between positive and negative feedback can be simply achieved by the design of the device matching ratios *B* and *C*.

The first advantage of the proposed VMA to be highlighted is the fact that Class-AB current peaks are generated at the output transistors only, with the known benefits in terms of low-power operation. Actually, the rest of the devices are operating in Class-A through the bias current sources $I_{\mathrm{tail}}$ and $I_{\mathrm{cmfb}}$, being the latter part of the CMFB loop, as explained at the end of this section.

The second feature is related to the Class-AB control itself. Under no output driving requirements, the VMA structure becomes fully balanced, and the resulting operating point at the output transistors is simply controlled by the 1:1 current mirror of Figure 7b fed with half $I_{\mathrm{tail}}$ (or $I_{\mathrm{cmfb}}$) tail current. On the contrary, when a strong differential input signal is present, the VMA core tends to modify its current-mirror topology up to the edge case of Figure 7c. Hence, the resulting dynamic ratio of this Class-AB current mirror gives the name to the variable-mirror amplifier. It can be shown that the maximum Class-AB peak current in all regions of operation, from weak to strong inversion, is found to be:

(3) $$\frac{I_{\max}}{I_{\mathrm{tail}}}=1+\frac{1}{C}\frac{AB}{A+B+C}\;$$

In practice, a good rule of thumb for optimizing the above Class-AB modulation index against parasitic poles is to choose:

(4) $$A\equiv B+C\;\frac{I_{\max}}{I_{\mathrm{tail}}}=1+\frac{B/C}{2}\;$$

**Figure 8:**
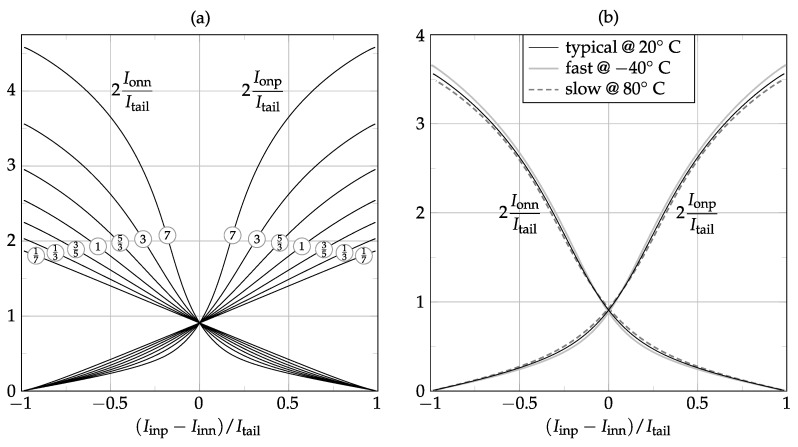
Simulated Class-AB current transfer curve for the VMA topology of Figure 7 at several B/C ratios using A≡B+C≐8 (**a**); and under technology and temperature combined corners for the B/C≡3 case (**b**).

Figure 8a shows how easily Class-AB modulation can be designed by just choosing different matching ratios between the *A*, *B* and *C* device multiplicities. Furthermore, since the Class-A operating point and the maximum Class-AB current are fully based on device matching only, the VMA operation exhibits very low sensitivity to both process and temperature variations, as verified in Figure 8b.

**Figure 9:**
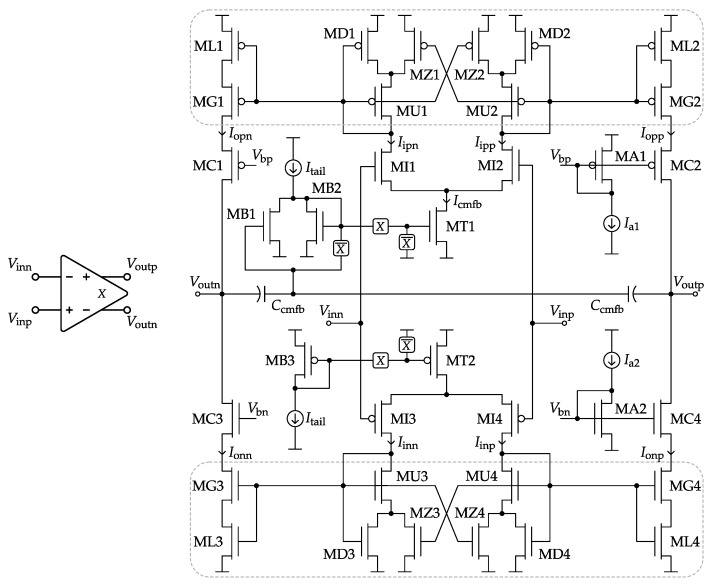
The switched-VMA circuit with cascode output and common mode feedback (CMFB) used in all of the ΔΣM SC integrators of Figure 5.

Last but not least, the VMA topology of Figure 7a can be still considered a single-stage amplifier, so it does not require internal frequency-compensation capacitors, with the consequent circuit area reduction, and it features short on-off settling times when operating as a switched-OpAmp. On the other hand, single stage amplifiers tends to suffer from low DC open-loop gain factors. In order to cope with this issue, the full switched-VMA (SVMA) circuit presented in Figure 9 includes cascode devices (MC1-4) at the output branches.

The switched-OpAmp operation required by the SC ΔΣM of Figure 5 is implemented in the proposed SVMA by means of the *X*-controlled switches at the output transistors of the tail current mirrors (MT1,2). Hence, regular amplification is performed for *X* high, while, during $\bar{X}$ phases, the SVMA is powered down by completely cutting off $I_{\mathrm{tail}}$ and $I_{\mathrm{cmfb}}$. Again, thanks to the single-stage nature of the SVMA and the lack of internal compensation capacitors, a high-speed power on-off switched-OpAmp operation can be easily achieved.

Concerning DC open-loop gain, output cascoding (MC) has been adopted to achieve the gain figures demanded by the target dynamic range. The suitable biasing of these stacked devices for a maximum output full-scale voltage is obtained from the optimum sizing of simple current mirrors (MA) [40].

The general design methodology to size the signal-path transistors of Figure 9 is as follows: the input differential pairs (MI1-4) are operated in moderate inversion to maximize the transconductance to current ratio; thanks to the output cascode devices’ MC, minimum channel length can be selected for the entire Class-AB core (MD1-4, MG1-4, ML1-4, MU1-4 and MZ1-4) in order to scale down internal parasitic capacitance; finally, the rule of thumb given in (4) is followed when designing the maximum Class-AB modulation index of the SVMA. For further details, a practical VMA circuit design example can be found in [11].

Finally, the mandatory CMFB loop for the fully-differential SVMA is implemented in Figure 9 through the control of the tail current $I_{\mathrm{cmfb}}$ of the NMOS input pair. For this purpose, a passive capacitive divider $C_{\mathrm{cmfb}}$ is attached to the differential output. Every time the SVMA is powered off, the ΔΣM SC network of Figure 5 together with MB devices precharge both $C_{\mathrm{cmfb}}$ elements. During SVMA on states, any common-mode output error is amplified and propagated to $I_{\mathrm{cmfb}}$ with the correct negative feedback supplied by the current subtraction between the MB1 and MB2 devices.

## 6. Simulation Results

**Figure 10:**
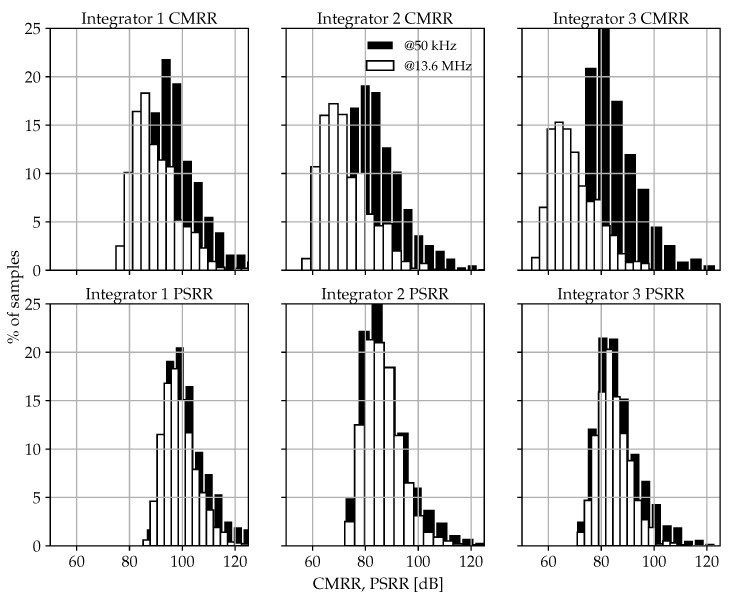
Simulated CMRR and PSRR deviations due to technology mismatch for each SVMA block of the SC ΔΣM of Figure 5.

Based on the complete amplifier circuit of Figure 9, each SVMA block of the SC ΔΣM of Figure 5 has been sized independently to achieve the performance requirements stated in Table 2. As an example, the electrical simulation results for the first-integrator SVMA are detailed in Table 3 to show its robustness against process and temperature deviations. Furthermore, it is worth noting the remarkable SVMA performance in terms of common-mode rejection ratio (CMRR) and power supply rejection ratio (PSRR) even under technology mismatch, as shown in Figure 10.

The full SC ΔΣM schematic of Figure 5, together with the optimized SVMA circuits, has been verified through electrical simulations using the transient-noise analysis of Cadence© Spectre© with the conservative-option profile. Each 13.28-kHz cycle of the input signal required about 6 h of computation time running a single thread on a 2.5-GHz 64-bit Intel© Xeon© E5-2640 CPU with 64-GB RAM. This large simulation-time figure illustrates the need for the ΔΣM behavioral model of Section 3 to perform optimization at the architecture level. Table 4 summarizes the 16-cycle simulation results for the full set of technology and temperature corners. As can be seen, the proposed ΔΣM circuit exhibits very low sensitivity to both process and temperature deviations. These results emphasize again the robustness of the proposed SVMA circuits and allow one to operate the full modulator without calibration.

**Figure 11:**
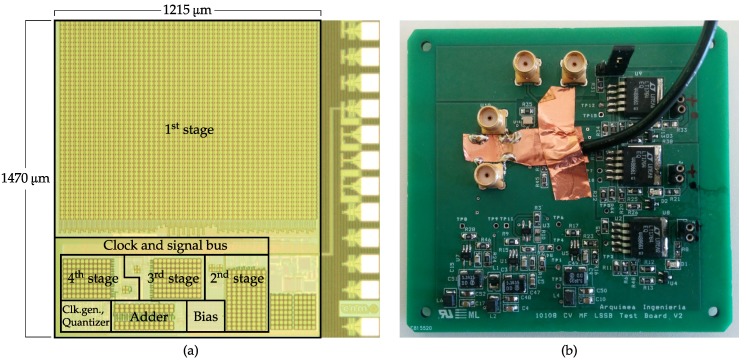
Microscope photography of the ΔΣM circuit in 0.18-μm CMOS technology (**a**); the core area is 1.8 mm^2^. Test PCB (**b**).

The post-layout simulation for the typical process and 27 °C temperature conditions returned a substantial drop of the ${\mathrm{SNDR}}_{\max}$ down to 103 dB. This degradation is mainly caused by two factors. First, and as can be seen from the chip photo of Figure 11, the differential signal path is routed following a local matching strategy instead of the classic global layout symmetry typical of fully-differential circuits. This choice sought to avoid signal coupling by minimizing the routing intersections, but it has been later demonstrated that its use introduces important parasitic asymmetries between the positive and negative paths of the differential signal. Second, and with less impact, convergence parameters were relaxed for the post-layout simulation in order to keep feasible CPU-time values.

## 7. Experimental Results

The ΔΣM was fabricated in a standard 0.18-μm 1-poly-6-metal CMOS technology with MIM (Metal-Insulator-Metal) capacitors, as shown in Figure 11, occupying an overall silicon area of 1.8 mm^2^ without the pads.

**Figure 12:**
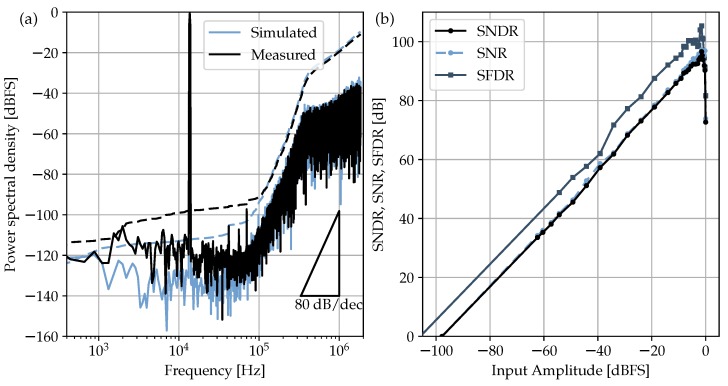
Measured and simulated ΔΣM output spectra for a −2-dB_FS_ and 13.28-kHz input. Equivalent SNDR values are 96.6 dB and 103 dB, respectively (**a**). Experimental ΔΣM dynamic range measured at 13.28 kHz (**b**).

The experimental characterization of Figure 12 was performed using the SRS DS360 function generator (Stanford Research Systems, Inc., Sunnyvale, CA, USA), which features low-noise ($1\;\mu V_{\mathrm{rms}}$) and low-distortion (below −100 dB) harmonic stimulus. A dedicated low-noise PCB was built to generate the three external voltage references ($V_{\mathrm{com}}$, $V_{\mathrm{fbp}}$ and $V_{\mathrm{fbn}}$) needed in the SC schematic of Figure 5. Regarding the clock signaling, a low-cost square-waveform generator was selected with 0.5-ns_rms_ jitter. The measured results show that the ΔΣM achieves a 96.6-dB peak SNDR, 105.3-dB peak SFDR and a 97-dB DR for 2.4-V_pp_ differential full scale and 50-kHz bandwidth. Compared to the simulation results of the previous section, experimental data return an extra 6-dB drop in dynamic range. Although the authors cannot give a unique reason for this extra loss, there are several effects that could be directly related to this degradation: first, substrate noise simulation was not available during the electrical design, and second, on-chip decoupling capacitors has not been added. In this sense, relying only on off-chip decoupling was revealed to be insufficient to avoid noise coupling with the critical signal paths. Unwanted and subtle interference couplings can also happen as soon as signals are extracted or inserted into the ASIC through wire bondings. Since wires propagating the clock and the digital output stream, both pulsed at 13.6MHz, are placed in the proximity of the very sensitive input signals, the possibility of coupling cannot be excluded even if a fully-differential configuration is adopted. A possible way to relieve this issue could be the use of the low-voltage-differential-signaling (LVDS) on the digital inputs/outputs. Here, standard digital pads provided with the design kit have been used; nevertheless, the target 16-bit specification is effectively achieved thanks to the two-bit safety margin adopted early in Section 3.

**Figure 13:**
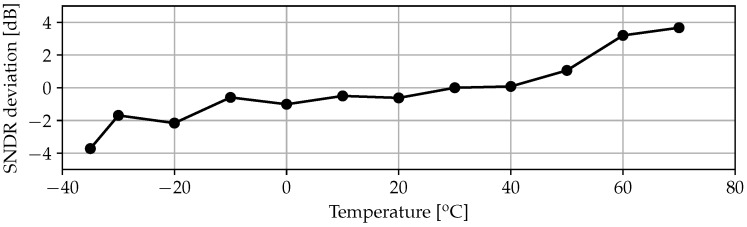
Measured SNDR deviation under temperature variations.

Concerning temperature sensitivity, the test has been performed by means of a Dycometal CCK-40/81 climatic chamber in the range between −35 °C and 70 °C ensuring an relative humidity (RH) between 50% and 60%. In principle the climatic chamber can operate in the wider range of (−40; 150) °C, but due to the physical connections with the external power sources and the input signal generator, the RH could not have been kept stable for temperatures higher than 70 °C. Results, shown in Figure 13, demonstrate deviations below ±4dB in the explored temperature range. This value, which resulted in being larger than the simulation estimations, can be ascribed to the thermal sensitivity of the PCB components, specially the reference voltage regulators employed for the modulator voltage references. In this case, the on-board voltage references are a scaled replica of the 1.8 V supply employing standard potentiometers followed by a unity-gain buffer (ICL7621 low-noise OpAmps from Intersil, Milpitas, CA, USA).

The whole ΔΣM circuit operates at the nominal supply voltage of 1.8 V and consumes 7.9 mW, which results in the following Schreier figure of merit:

(5) $${\mathrm{FOM}}_{S}=\mathrm{SNDR}+10\log\left(\frac{\mathrm{BW}}{P_{D}}\right)=164.6\;\mathrm{dB}.$$

Table 5 shows an extensive comparison of the state-of-the-art high-resolution CMOS ADCs [13,14,15,16,17,18,19,20,21,22,23] including the measured ${\mathrm{SNDR}}_{\max}$ and the corresponding ${\mathrm{FOM}}_{S}$. References are classified according to the requirements of supply bootstrapping and analog calibration.

As can be noticed, this work is well positioned with respect to existing references operating at the nominal supply voltage of the CMOS technology and not making use of any kind of analog calibration or digital post-compensation. Furthermore, the work presented here features the highest experimental ${\mathrm{FOM}}_{S}$ if resistor-less restriction is also of concern. It is worth highlighting here that the design of the SVMAs was probably too conservative for this first-time usage in SC ΔΣMs, so further improvements in ${\mathrm{FOM}}_{S}$ should be in principle reachable with the proposed solution.

While the FOM_s_ clearly lumps the speed-resolution-power trade-offs in one single number, it ignores other design trade-offs, like total chip area and feasible connectivity in a multiplexed channel system like the one needed in an IR imager. For the specific application depicted in Figure 1, the authors propose a companion performance parameter taking into account the following aspects: (i) the power budget for the whole ADC array should not exceed 300 mW; (ii) the maximum number of ADCs should be limited to 36 due to the physical implementability of the interconnections with the detector array. These requirements descend directly from the specific system packaging technique, which contemplates the ASIC to be mounted underneath the IR imager via the interposer. In order to minimize area overhead and thermal mass, the ASIC is bare-die bonded to the interposer, which in turn features dedicated metal paths for heat flow regulation. Now, under the above-mentioned limitations, the effective frame rate (EFR) is calculated as follows:

(6) $$\mathrm{EFR}=\min\left(\frac{300}{P_{D}\left(\mathrm{mW}\right)},36\right)\cdot \frac{f_{\mathrm{Nyquist}}}{n_{\mathrm{FPA}}}$$

where $n_{\mathrm{FPA}}$ is the number of the pixels of the detector, and $f_{\mathrm{Nyquist}}$ is the ADC speed. For the considered application, a minimum dynamic range of 16 ENOB is required to comply with state-of-the-art IR imagers; moreover, in order to provide a fair comparison with the state-of-the-art of Table 5, $f_{\mathrm{Nyquist}}$ of the designs exceeding 16 ENOB is calculated extending their bandwidth according to the following correction formula:

(7) $$f_{\mathrm{Nyquist}}=2\times \mathrm{Corrected}\;\mathrm{Bandwidth}=2\times \mathrm{Declared}\;\mathrm{Bandwidth}\times 10^{\frac{\mathrm{ENOB}\;\mathrm{Excess}}{20(N+0.5)}}$$

being *N* the modulator order. This formula descends directly from the Signal-to-Quantization-Noise Ratio (SQNR) estimation, thus assuming that the extension of the modulator bandwidth explores a region where the quantization noise starts to be dominant over the thermal noise as typically occurs in power-optimized ΔΣM designs. Using Equations (6) and (7), the comparison of Table 6 is obtained, showing that the this work doubles the EFR with respect to the state-of-the-art.

## 8. Conclusions

A 96.6-dB-peak-SNDR and 50-kHz-bandwidth SC ΔΣM implemented in a standard 0.18-μm CMOS technology has been presented. Operating at the nominal supply voltage of 1.8 V and consuming 7.9 mW, it reaches a 164.6-dB Schreier FOM without making use of any clock bootstrapping, analog calibration, nor digital post-compensation. These design decisions have been taken to minimize any failure risk associated with devices’ gate-oxide overstress and added circuitry failures. The performances of the presented modulator are largely achieved thanks to a combination of a number of techniques both at the architectural and at the circuital level: single-bit feedback DAC, moderate-high OSR and matching-ratio-based design from the SC architecture syncretically blend with the novel power-efficient Class-AB switched-VMAs to give a ΔΣM with very low sensitivity to both the CMOS process and temperature deviations. The authors envision room for further power optimization through a more accurate input sampler sizing in the next generation of this ASIC; nevertheless, the application of the proposed converter to the 2048×2048 IR imager for space instrumentation allows for an effective frame rate exceeding 50fps when practical area and connectivity constraints are taken into account. This figure finally resulted in a two-fold improvement with respect to the state-of-the-art.

## Figures and Tables

**Table 1:** Capacitor sizing in pF for the SC ΔΣM of  Figure 5.

Capacitance	Value	Capacitance	Value	Capacitance	Value
$C_{\mathrm{fb}}$	21.16			$C_{\mathrm{ff}0}$	0.92
$C_{s1}$	42.32	$C_{i1}$	211.6	$C_{\mathrm{ff}1}$	0.92
$C_{s2}$	3.68	$C_{i2}$	9.2	$C_{\mathrm{ff}2}$	0.92
$C_{s3}$	0.92	$C_{i3}$	9.2	$C_{\mathrm{ff}3}$	0.92
$C_{s4}$	0.92	$C_{i4}$	9.2	$C_{\mathrm{ff}4}$	1.84

**Table 2:** Specifications for the SC ΔΣM amplifiers of  Figure 5.

Parameter	SVMA1	SVMA2	SVMA3,4	Units
$C_{\mathrm{load}}$	53.4	4.47	2.68	pF
$\beta_{\mathrm{fb}x}$	0.77	0.71	0.91	-
$t_{\mathrm{sett}}\;\left(0.035\%\right)$	<28	<28	<28	ns

**Table 3:** Simulated performance of SVMA1 under process and temperature corners.

Parameter	Typical 20 °C	Fast −40 °C	Slow 80 °C	Units
*G*(DC)	74.1	74.7	73.9	dB
$I_{\mathrm{tail}}$	1	1	1	mA
$I_{\max}$	7.41	7.8	7.1	mA
SR	139	146	133	V/µs
GBW	221	250	206	MHz
$\mathrm{PM}\;(\beta_{\mathrm{fb}})$	55.8	48.9	62.8	°
$t_{\mathrm{sett}}\;\left(0.035\%\right)$	16.41	14.24	19.57	ns

**Table 4:** ΔΣM simulated SNDR at −2-dB_FS_ under process and temperature corners.

	Slow	Typical	Fast	
−40 °C	110.2	109.2	111.4	dB
27 °C	110.6	111.3	110.7	dB
80 °C	110.7	109.3	109.6	dB

**Table 5:** Comparison of CMOS ADCs with SNDR exceeding 90 dB. SAR, successive approximation register.

	[13]	[14]	[15]	[16]	[17]	[18]	[19]	[20]	[21]	[22]	[23]	This Work
Technology (nm)	350	180	28	350	250	180	180	160	180	180	180	180
Architecture	ΔΣ	ΔΣ	ΔΣ	ΔΣ	ΔΣ	ΔΣ	ΔΣ	SAR + ΔΣ	ΔΣ	ΔΣ	ΔΣ	ΔΣ
Modulator order	2	2	2	5	4	2	5	2	3	3	3	4
Circuit technique	RC + SC	SC	RC	SC	SC	RC + SC	RC + Gm-C	SC	RC	SC	RC	SC
Supply voltage (V)		0.7	1, 3.3	5		3.3	1.8	1.8		5	1.8	1.8
Diff. full scale (V_pp_)					6.6	5.7	1.4	1.8		4.4		2.4
Sampling rate (MS/s)	6.14	5	24	5.12	20	6.14	41.7	0.05	57.5	0.15	6.144	13.6
Bandwidth (kHz)	20	25	24	20	1000	20	200	0.0125	600	0.1	24	50
Supply power (mW)	18	0.87	1.13	55	475	37	210	0.0063	21	0.505	0.28	7.9
Area (mm^2^)	0.82	2.16	0.022	5.6	20.2	0.65	6	0.38	0.99	0.8	1.33	1.8
DR (dB)	106	100	100.6	111	103	102	98				103.6	97
${\mathrm{SFDR}}_{\max}$ (dB)			102.6						90	100.8	107.6	105.3
${\mathrm{SNDR}}_{\max}$ (dB)	97	95	98.5	105		95	90			100.6	98.5	96.6
${\mathrm{FOM}}_{S}$ (dB)	157.5	169.6	171.8	160.6	166.2 ^*^	152.3	149.8	182.8 ^*^	164.6 ^*^	153.6	177.8	164.6
Bootstrap-free	✓			✓	✓	✓	✓	✓	✓	✓	✓	✓
Calibration-free		✓	✓	✓	✓	✓	✓	✓	✓	✓	✓	✓

* FOM_s_ values not obtained from SNDR_max_, but from DR, SNR or SFDR figures. Circuit technique acronyms: RC, resistor-capacitor; SC, switched-capacitor; Gm-C, transconductor-capacitor.

**Table 6:** Effective number of frames per minute from Equation (6) with $n_{\mathrm{FPA}}=2048\times 2048$. EFR, effective frame rate.

	[13]	[15]	[16]	[20]	[22]	[23]	This Work
EFR (f/min)	9.16	25.01	2.9	0.01	0.11	24.93	51.42
Number of ADCs	16	36	5	36	36	36	36
